# Supplementary material for: Connecting laboratory behavior to field function through stable isotope analysis
Source: PeerJ. 2016 Apr 11;4:e1918. doi: 10.7717/peerj.1918 (PMC4830243; doi:10.7717/peerj.1918)
Supplement: Supplemental Information 1 [file peerj-04-1918-s002.pdf]

Supplementary material for "Connecting Laboratory Behavior to Field Function through Stable Isotope Analysis"

Glon, Mael G.*, Larson, Eric R.† & Pangle, Kevin L.*

*Department of Biology, Central Michigan University, Mount Pleasant, Michigan 48859

†Department of Natural Resources and Environmental Sciences, University of Illinois, Urbana, Illinois 61801

Correspondence:

Mael Glon

Brooks Hall 217

Central Michigan University

Mt Pleasant, MI 48859

glon1mg@cmich.edu

Page Content

Page	Content
3	I. Crayfish morphometrics
4	Table S1. Crayfish Morphometrics
5	II. Alternative comparisons of dominance and trophic position
8	Figure S1. Scatterplot of crayfish carapace length and dominance scores
9	Figure S2. Scatterplot of the residuals from dominance scores and carapace length regression and calculated trophic position
10	Figure S3. Scatterplot of crayfish dominance scores and δ^{15}N signatures
11	III. Analysis of the relationship between percent reliance of crayfish on the snail primary production pathway and dominance
13	Figure S4. Scatterplot of crayfish dominance scores and percent reliance on snail primary production
14	IV. References

I. Crayfish morphometrics

Size influences the outcome of crayfish agonistic trials (Rubenstein & Hazlett, 1974; Bergman & Moore, 2003); therefore, to better understand what intrinsic factors might be affecting the results of our agonistic assays, we used digital calipers to measure carapace length (CL; from the tip of the rostrum to the posterior edge of the carapace), chelae width (at the widest point of the palm), and chelae length (from the attachment of the carpus and the propodus to the most distal point of the fixed finger) to the nearest hundredth of a mm. We used a digital balance to measure mass to the nearest hundredth of a gram (Table S1). Prior to weighing, we dabbed all crayfish dry for 10 seconds with a paper towel.

Table S1. Crayfish morphometrics.

Measurement	Mean	Standard Deviation	Minimum	Maximum
Carapace length (mm)	25.38	1.26	23.41	27.53
Chelae length (mm)	17.38	1.70	14.05	21.36
Chelae width (mm)	7.20	0.85	5.02	8.31
Mass (g)	5.06	0.78	3.7	6.5

II. Alternative comparisons of dominance and trophic position

Body size is a factor that strongly influences the outcome of agonistic encounters in crayfish, with larger individuals generally being more dominant (Bovbjerg, 1953; Rubenstein & Hazlett, 1974; Bergman & Moore, 2003). We used as small of a crayfish size range as logistically possible, but the difference between our largest and smallest study organisms was still 4.12 mm carapace length (Table S1). Despite this, most paired agonistic interaction trials were between more closely size-matched crayfish (mean ± standard deviation; 1.44 ± 1.15 mm carapace length). Regardless, we sought to determine if dominance scores might better correspond with the trophic positions of our crayfish if we corrected for the role of size differences in determining outcomes of agonistic interactions. We did not correct for potential ontogenetic effects of crayfish size on trophic position (Bondar *et al.*, 2005; Larson, Olden & Usio, 2010), because we found no significant relationship between crayfish carapace length (Table S1) and trophic position ($y = 0.002x + 2.27$, $R^2 = 0.001$, $F_{1,38} = 0.02$, $p = 0.88$). However, as we anticipated, there was a significant relationship between crayfish carapace length and mean dominance score ($y = 11.503x - 261.971$, $R^2 = 0.26$, $F_{1,38} = 13.03$, $p < 0.001$; Figure S1). Yet, when we corrected for the effect of crayfish size on dominance by regressing residuals of the preceding analysis against trophic position, we still did not find a significant relationship, consistent with our main text conclusion ($y = 0.00x + 2.34$, $R^2 = 0.01$, $F_{1,38} = 0.54$, $p = 0.47$; Figure S2). The lack of a relationship between dominance and trophic position is therefore conserved even when accounting for the potential influence of crayfish size on dominance.

Carapace length is the most commonly used size metric for crayfish; however, chelae size has been shown to dictate success in agonistic encounters and may be a better measure of dominance in crayfish (Garvey & Stein, 1993). We therefore ran two additional iterations of the

analysis presented above, using chelae length and width instead of carapace length. We found significant relationships between mean dominance scores and both chelae length (y = 9.125x – 128.686, R^2 = 0.29, $F_{1,38}$ = 15.72, p < 0.001) and chelae width (y = 16.040x – 85.562, R^2 = 0.23, $F_{1,38}$ = 11.07, p = 0.002). Yet again, regressing residuals from the chelae length or width and dominance score analyses against trophic position did not change our main text conclusion that that dominance and trophic position are unrelated (chelae length residuals vs trophic position: y = 0.0002x – 2.34, R^2 = 0.001, $F_{1,38}$ = 0.04, p = 0.85; chelae width residuals vs trophic position: y = -0.0001x – 2.34, R^2 = 0.01, $F_{1,38}$ = 0.02, p = 0.89).

The use of isotopic mixing models, applied here as a step in calculating trophic position (Post, 2002), is dependent on a number of assumptions. For example, stream and river ecosystems can have extremely high spatiotemporal variation in the $\delta^{13}C$ and $\delta^{15}N$ values of sources of primary production owing to a number of factors (Fry & Sherr, 1984; Finlay, 2001; Trudeau & Rasmussen, 2003). Accordingly, we followed convention in using primary consumers rather than primary producers in mixing model calculations of trophic position, as long-lived organisms like mussels or snails can integrate and correct for this variability (Post, 2002; Cabana & Rasmussen, 1996). However, we cannot exclude that our field sampling of primary consumer endpoints for our mixing model could have missed some such variability inherent to heterogeneous lotic ecosystems, and our collection of potential prey resources concurrent with crayfish consumers does not necessarily reflect isotopic values of prey items for *Orconectes rusticus* over preceding weeks or months (Moore & Semmens, 2008). Another assumption of mixing models is that constant discrimination factors can be used for each trophic step and between different taxonomic groups and diet items. However, discrimination factors can vary across taxa, diets, and tissues used (e.g., Stenroth *et al.*, 2006; Caut, Angulo & Courchamp,

2009; Phillips *et al*., 2014), and consequently may misrepresent trophic position of a focal organism (Bond & Diamond, 2011). Due to the potential vulnerability of our model to the preceding assumptions, we also conducted a simpler analysis using crayfish dominance scores and unaltered $\delta^{15}N$ values to determine if our results were dependent on our specific trophic position calculations. Doing so did not alter our overall nonsignificant result and conclusion ($y = 0.002x + 11.04$, $R^2 = 0.03$, $F_{1,38} = 1.29$, $p = 0.26$; Figure S3). We therefore conclude that our result of a lack of relationship between crayfish dominance in the laboratory and trophic position in the field is robust to our measures of both crayfish dominance and trophic position.

Figure S1.

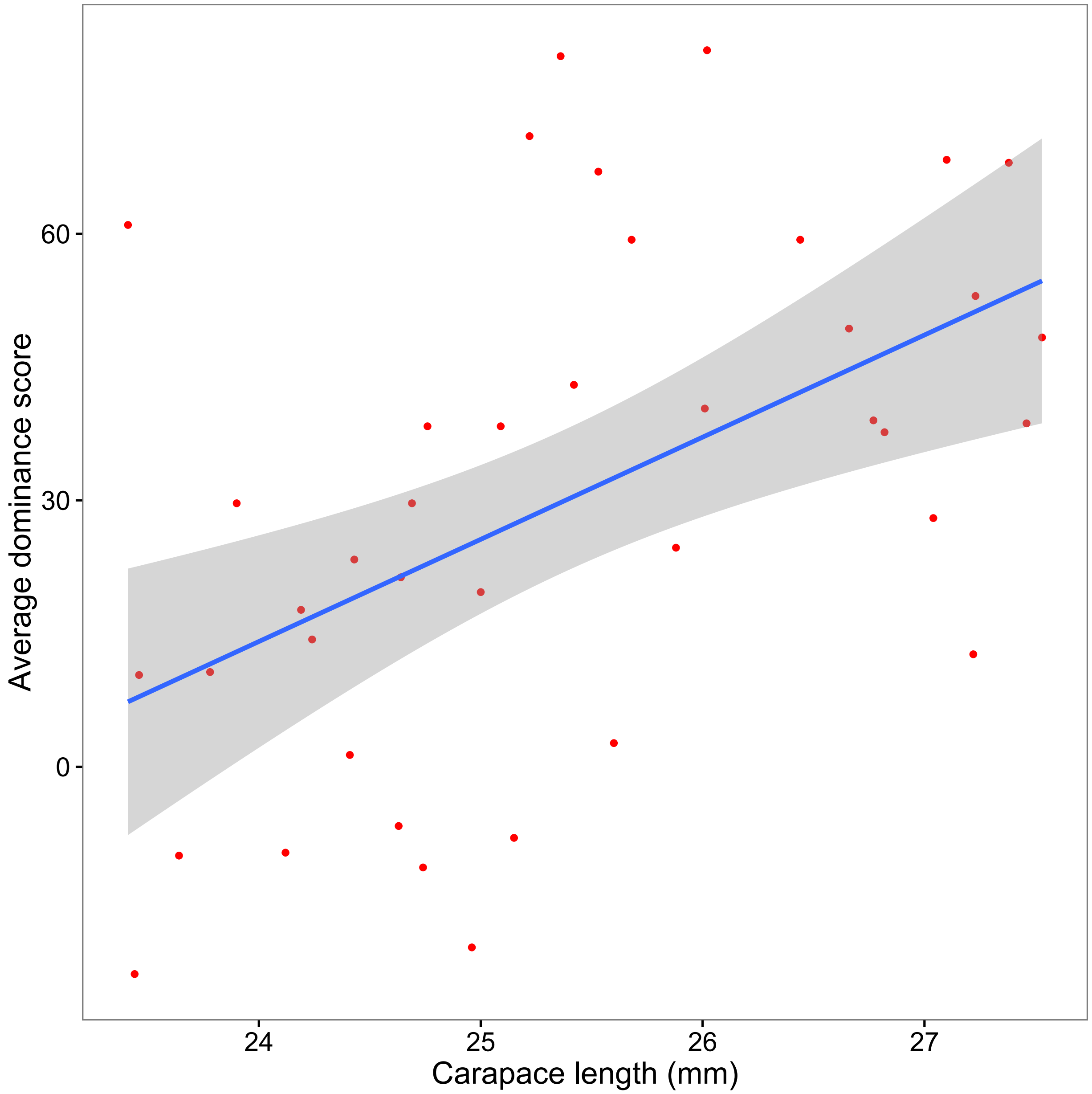

Figure S1. Scatterplot (with 95% CI) showing significant relationship between crayfish carapace length and dominance score from behavioral assays ($y = 11.503x - 261.971$, $R^2 = 0.26$, $F_{1,38} = 13.03$, $p < 0.001$).

Figure S2.

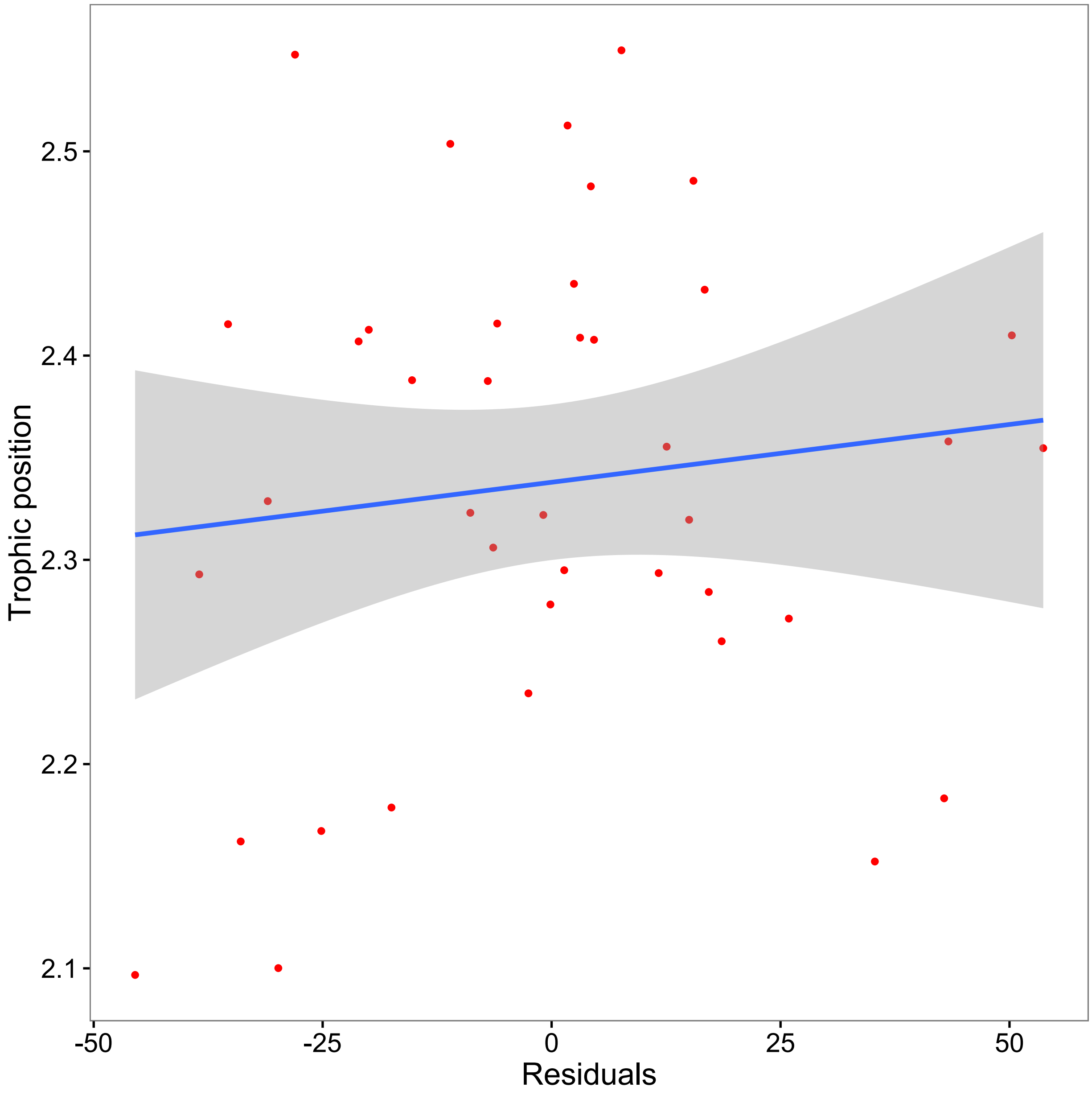

Figure S2. Scatterplot (with 95% CI) of the residuals from crayfish dominance and carapace length regression against calculated trophic position ($y = 0.001x + 2.34$, $R^2 = 0.01$, $F_{1,38} = 0.54$, $p = 0.47$).

Figure S3.

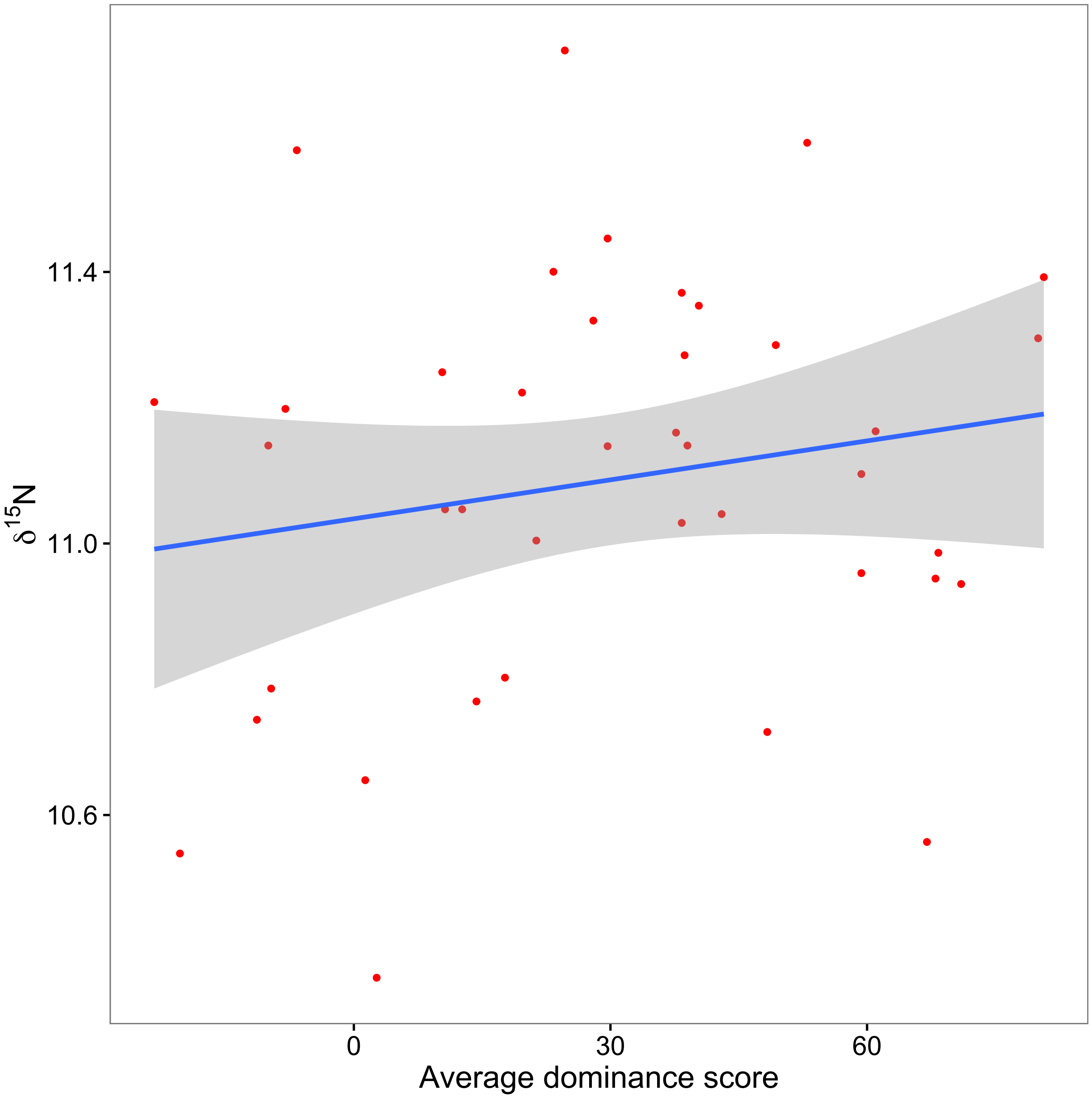

Figure S3. Scatterplot (with 95% CI) of crayfish dominance scores and $\delta^{15}N$ signatures (y = 0.002x + 11.04, $R^2 = 0.03$, $F_{1,38} = 1.29$, p = 0.26)

III. Analysis of the relationship between percent reliance of crayfish on the snail primary production pathway and dominance

Our mixing model calculations revealed variation in the percent reliance of *O. rusticus* from the Chippewa River on food resources represented by the two primary consumer endpoints used in our study. Specifically, crayfish relied more on the isotopically-enriched primary production represented by snails (mean ± standard deviation, 67.6 ± 11.3%) than on the isotopically-depleted primary production represented by mussels (32.4 ± 11.3 %; Figure 2). In lotic systems, the isotopic values of freshwater mussels generally reflect those of a broad range of potential sources of primary production including terrestrial detritus and phytoplankton from upstream lentic systems (Raikow & Hamilton, 2001; Cole & Solomon, 2002). Therefore, reliance of our crayfish on the mussel endpoint may reflect dependence of *O. rusticus* on terrestrial detritus, in part because we do not anticipate high reliance of crayfish on phytoplankton (Stenroth *et al.*, 2006). Conversely, reliance of *O. rusticus* on snails likely represents use of benthic algae as a basal resource, particularly given the generally low trophic positions of the crayfish in our study. Benthic algae or other autochthonous production in freshwater ecosystems may be a higher quality diet item than terrestrial detritus (Finlay, 2001; Brett *et al.*, 2009), and consequently, we hypothesized that more dominant crayfish in our behavioral assays might show greater dependence on algal or snail resources than the alternative resources represented by freshwater mussels. In order to test this hypothesis, we ran a regression between percent reliance on the snail endpoint of our mixing model and our dominance assay scores, as previously analyzed for trophic position (see main text and above). Again, we did not find a significant relationship between crayfish dominance and diet ($y = 0.02x + 66.98$, $R^2 = 0.002$, $F_{1,38} = 0.09$, $p = 0.76$; Figure S4), further supporting our conclusion that results of *ex situ*

laboratory behavioral trials do not necessarily translate to the hindcasted *in situ* ecology of these same individual organisms.

Figure S4.

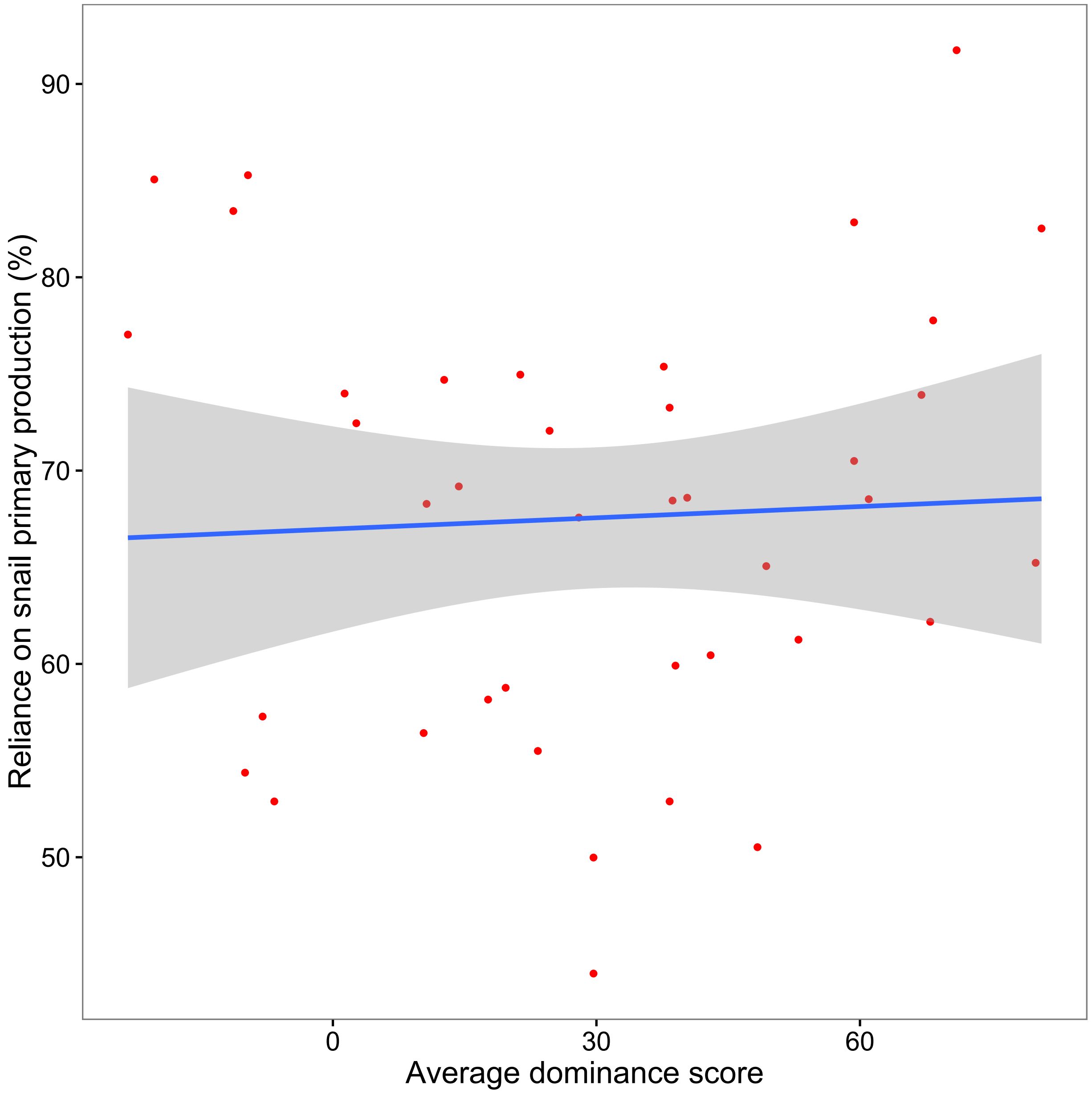

Figure S4. Scatterplot (with 95% CI) of mean assay dominance score for each crayfish over three agonistic assays and percent reliance on the snail primary production pathway ($y = 0.02x + 66.98$, $R^2 = 0.002$, $F_{1,38} = 0.09$, $p = 0.76$).

IV. References

1. Bergman DA, Moore PA. 2003 Field observations of intraspecific agonistic behavior of two crayfish species. *The Biological Bulletin* **205**, 26-35. (doi:10.2307/1543442)
2. Bond AL, Diamond AW. 2011 Recent Bayesian stable-isotope mixing models are highly sensitive to variation in discrimination factors. *Ecological Applications* **21**, 1017-1023.
3. Bondar CA, Bottriell K, Zeron K, Richardson JS. 2005 Does trophic position of the omnivorous signal crayfish (*Pacifastacus leniusculus*) in a stream food web vary with life history stage or density? *Canadian Journal of Fisheries and Aquatic Sciences* **62**, 2632-2639. (doi: 10.1139/f05-167)
4. Bovbjerg RV. 1953 Dominance order in the Crayfish *Orconectes virilis* (Hagen). *Physiological Zoology* **26**, 173-178.
5. Brett MT, Kainz MJ, Taipale SJ, Seshan H. 2009 Phytoplankton, not allochthonous carbon, sustains herbivorous zooplankton production. *Proceedings of the National Academy of Sciences of the United States of America* **106**, 21197-21201.
6. Cabana G, Rasmussen JB. 1996 Comparison of aquatic food chains using nitrogen isotopes. *Proceedings of the National Academy of Sciences of the United States of America* **93**, 10844-10847.
7. Caut S, Angulo E, Courchamp F. 2009. Variation in discrimination factors ($\Delta^{15}N$ and $\Delta^{13}C$): the effect of diet isotopic values and applications for diet reconstruction. *Journal of Applied Ecology* **46**, 443-453. (doi:10.1111/j.1365-2664.2009.01620.x)
8. Cole JJ, Solomon CT. 2012 Terrestrial support of zebra mussels and the Hudson River food web: A multi-isotope, Bayesian analysis. *Limnology and Oceanography.* **57**, 1802-1815. (doi:10.4319/lo.2012.57.6.1802)

9. Finlay JC. 2001 Stable-carbon-isotope ratios of river biota: implications for energy flow in lotic food webs. *Ecology* **82**, 1052-1064.(doi:10.1890/0012-9658(2001)082[1052:SCIROR]2.0.CO;2)
10. Fry B, Sherr EB. 1984 $\Delta^{13}C$ measurements as indicators of carbon flow in marine and freshwater ecosystems. *Contributions in Marine Science* **27**, 13-47. (doi:10.1007%2F978-1-4612-3498-2_12)
11. Garvey JE, Stein RA. 1993 Evaluating how chela size influences the invasion potential of an introduced crayfish (*Orconectes rusticus*). *The American Midland Naturalist* **129**, 172-181. (doi:10.2307/2426446)
12. Larson ER, Olden JD, Usio N. 2010 Decoupled conservatism of Grinnellian and Eltonian niches in an invasive arthropod. *Ecosphere* **1**, 1-13. (doi: http://10.1890/ES10-00053.1)
13. Moore JW, Semmens BX. 2008 Incorporating uncertainty and prior information into stable isotope mixing models. *Nature* **11**, 470-480. (doi:10.1111/j.1461-0248.2008.01163.x)
14. Phillips DL, Inger R, Bearhop S, Jackson AL, Moore JW, Parnell AC, Semmens BX, Ward EJ. 2014 Best practices for use of stable isotope mixing models in food web studies. *Canadian Journal of Zoology* **92**, 823-835. (doi: http://10.1139/cjz-2014-0127)
15. Post DM. 2002 Using stable isotopes to estimate trophic position: models, methods, and assumptions. *Ecology* **83**, 703-718. (doi:10.1890/0012-9658(2002)083[0703:USITET]2.0.CO;2)
16. Raikow DF, Hamilton SK. 2001 Bivalve diets in a Midwestern U.S. stream: A stable isotope enrichment study. *Limnology and Oceanography* **46**, 514-422. (doi:10.4319/lo.2001.46.3.0514)

17. Roth BM, Hein CL, Vander Zanden MJ. 2006 Using bioenergetics and stable isotopes to assess the trophic role of rusty crayfish (*Orconectes rusticus*) in lake littoral zones. *Canadian Journal of Fisheries and Aquatic Sciences* **63**, 335-344. (doi:10.1139/f05-217)

18. Rubenstein DI, Hazlett BA. 1974 Examination of the agonistic behavior of the crayfish *Orconectes virilis* by character analysis. *Behavior* **50**, 193-216. (doi:10.2307/4533609?ref=no-x-route:1dae0e260ae10f0d6415e334220ef8a2)

19. Stenroth P, Homqvist N, Nyström P, Berglund O, Larsson P, Granéli W. 2006 Stable isotopes as an indicator of diet in omnivorous crayfish (*Pacifastacus leniusculus*): the influence of tissue, sample treatment, and season. *Canadian Journal of Fisheries and Aquatic Sciences* **63**, 821-831. (doi:10.1139/f05-265)

20. Trudeau V, Rasmussen JB. 2003 The effect of water velocity on stable carbon and nitrogen isotope signatures of periphyton. *Limnology and Oceanography* **48**, 2194-2199. (doi:10.4319/lo.2003.48.6.2194)
